# Patients ‘ perspectives on bone replacement materials in a German university hospital setting

**DOI:** 10.1186/s12938-023-01147-2

**Published:** 2023-08-28

**Authors:** Ana Prates Soares, Heilwig Fischer, Vincenzo Orassi, Max Heiland, Sara Checa, Katharina Schmidt-Bleek, Carsten Rendenbach

**Affiliations:** 1 Department of Oral and Maxillofacial Surgery, Charité—Universitätsmedizin Berlin, Corporate Member of Freie Universität Berlin, and Humboldt-Universität zu Berlin, and Berlin Institute of Health, Berlin, Germany; 2 Julius Wolff Institute for Biomechanics and Musculoskeletal Regeneration, Berlin Institute of Health at Charité—Universitätsmedizin Berlin, Berlin, Germany; 3 Centrum für Muskuloskeletale Chirurgie, Charité—Universitätsmedizin Berlin, Corporate Member of Freie Universität Berlin, and Humboldt-Universität zu Berlin, and Berlin Institute of Health, Berlin, Germany; 4 Institute of Health (BIH) Biomedical Innovation Academy, BIH Charité Clinician Scientist Program, Berlin Institute of Health at Charité—Universitätsmedizin Berlin, Berlin, Germany; 5 Berlin Institute of Health (BIH) Centre for Regenerative Therapies (BCRT), Berlin Institute of Health at Charité—Universitätsmedizin Berlin, Berlin, Germany

**Keywords:** Surveys and questionnaires, Bone replacement material, Patient Engagement

## Abstract

**Background:**

The challenges in developing new bone replacement materials and procedures reside not solely in technological innovation and advancement, but also in a broader patient therapy acceptance. Therefore, there is a need to assess patients’ perspectives on the materials and approaches in use as well as the ones being developed to better steer future progress in the field.

**Methods:**

A self-initiating cross-sectional questionnaire aimed at people seeking treatment at the university hospital environment of Charité Berlin was formulated. The survey contained 15 close-ended questions directed toward the participant’s epidemiological profile, willingness, acceptance, and agreement to receive different bone replacement materials, as well as, worries about the post-surgical consequences that can arise post bone replacement surgery. Descriptive and categorical analysis was performed to compare the observed number of subjects, their profile and each related response (Pearson’s chi-square test or Fischer’s test, *p* < 0.05).

**Results:**

A total of 198 people engaged with the questionnaire, most of them Millennials. Overall patients trusted scientifically developed biomaterials designed for bone replacement, as demonstrated by their willingness to participate in a clinical trial, their acceptance of alloplastic materials, and the none/few worries about the presence of permanent implants. The data revealed the preferences of patients towards autologous sources of cells and blood to be used with a biomaterial. The data have also shown that both generation and education influenced willingness to participate in a clinical trial and acceptance of alloplastic materials, as well as, worries about the presence of permanent implants and agreement to receive a material with pooled blood and cells.

**Conclusion:**

Patients were open to the implantation of biomaterials for bone replacement, with a preference toward autologous sources of blood and/or tissue. Moreover, patients are concerned about strategies based on permanent implants, which indicates a need for resorbable materials. The knowledge gained in this study supports the development of new bone biomaterials.

**Supplementary Information:**

The online version contains supplementary material available at 10.1186/s12938-023-01147-2.

## Introduction

Bone replacement materials are used to support and promote the regeneration of bone defects. Traditionally, bone replacements are divided into autograft (bone graft taken from another site of the same patient), allograft (bone obtained from human donors), xenograft (bone taken from donors from another species), and alloplastic (synthetic materials chemically similar to bone). To stabilize these materials and integrate them into the bone, fixation plates, and screws can be used, mostly composed of inert metals or biodegradable alloys. These approaches have several limitations including the limited bone availability, the patient’s immune response, and the need for a second surgery. To overcome these limitations, the modern idea of tissue-engineered bone materials was brought about in the mid-1980s [1, 2]. The general modern concept of tissue engineering and regenerative medicine is to produce functional structures or scaffolds that will support tissue regeneration during patient treatment. Since then, many different biomaterials for bone replacement have been proposed and tested all made of different materials (ceramics, polymers, metals, etc.) and with different designs, at times using human cells or tissue as part of the material [3]. Although there are many options, there is still no ideal material for every clinical case, making the material choice a part of the surgical plan.

In recent years, there has been an increased amount of available information about medical treatments, materials, and procedures for the patient, which has directly enabled them to ask about or suggest different approaches for their cases. Patient involvement in the decision-making process in orthopedic surgery is being emphasized as part of a shared decision-making treatment concept [4, 5]. This concept has as its objective to empower patients in their search for health and has proven to reduce healthcare costs and improve the quality of care [6]. Furthermore, from the translational side of newly developed materials, the new health technology assessment (HTA) process requires the participation of patients and patient groups as stakeholders from conception to the application of new technology [7]. The new HTA aims to assess patients’ actual needs and the impact that the technology may have on their lives. Therefore, assessing patients’ perspectives and concerns about bone replacement materials and procedures can help steer new bone replacement material development.

The present work aimed to survey and assess patients´ personal views about the use of different bone replacement materials and post-surgery worries. This study was designed to support and improve the development of future treatment approaches so they can better address potential patients’ fears and needs.

## Results

**Fig. 1:**
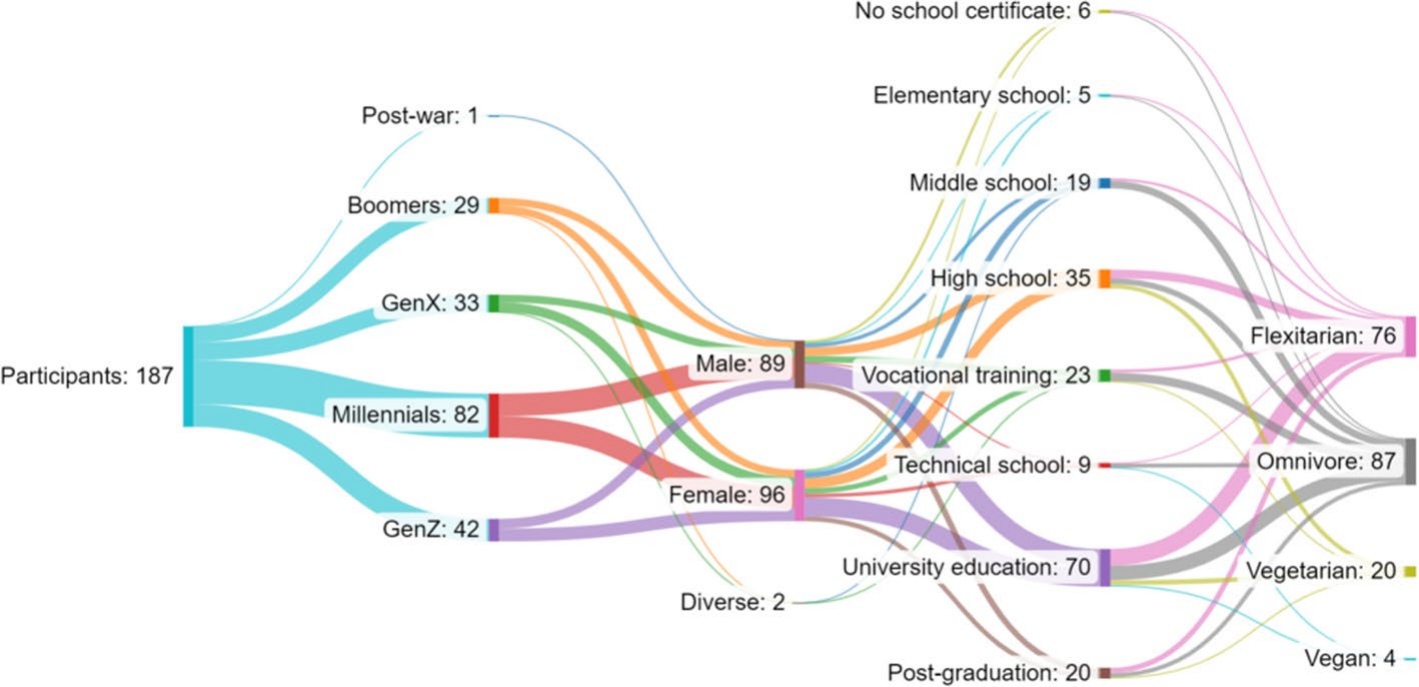
Sankey diagram displaying the participants’ distribution into generation, gender, schooling levels, and nutritional choices (Created using SankeyMATIC: https://sankeymatic.com/)

The respondents’ profile was mixed (Fig. 1) with a higher presence of Millennials (43%) and people with university-level education (37%). There was a similar frequency of females (51%) and males (47%), as well as omnivores (46%) and flexitarians (40%). Once data were dichotomized, it was revealed that the occurrence of flexitarian/vegetarian/vegan eaters or omnivores was dependent on gender (Chi-square, *p* = 0.0011), and a higher amount of people who identified as male were omnivores. No other statistical differences were found in the population’s epidemiological characteristics.

**Fig. 2:**
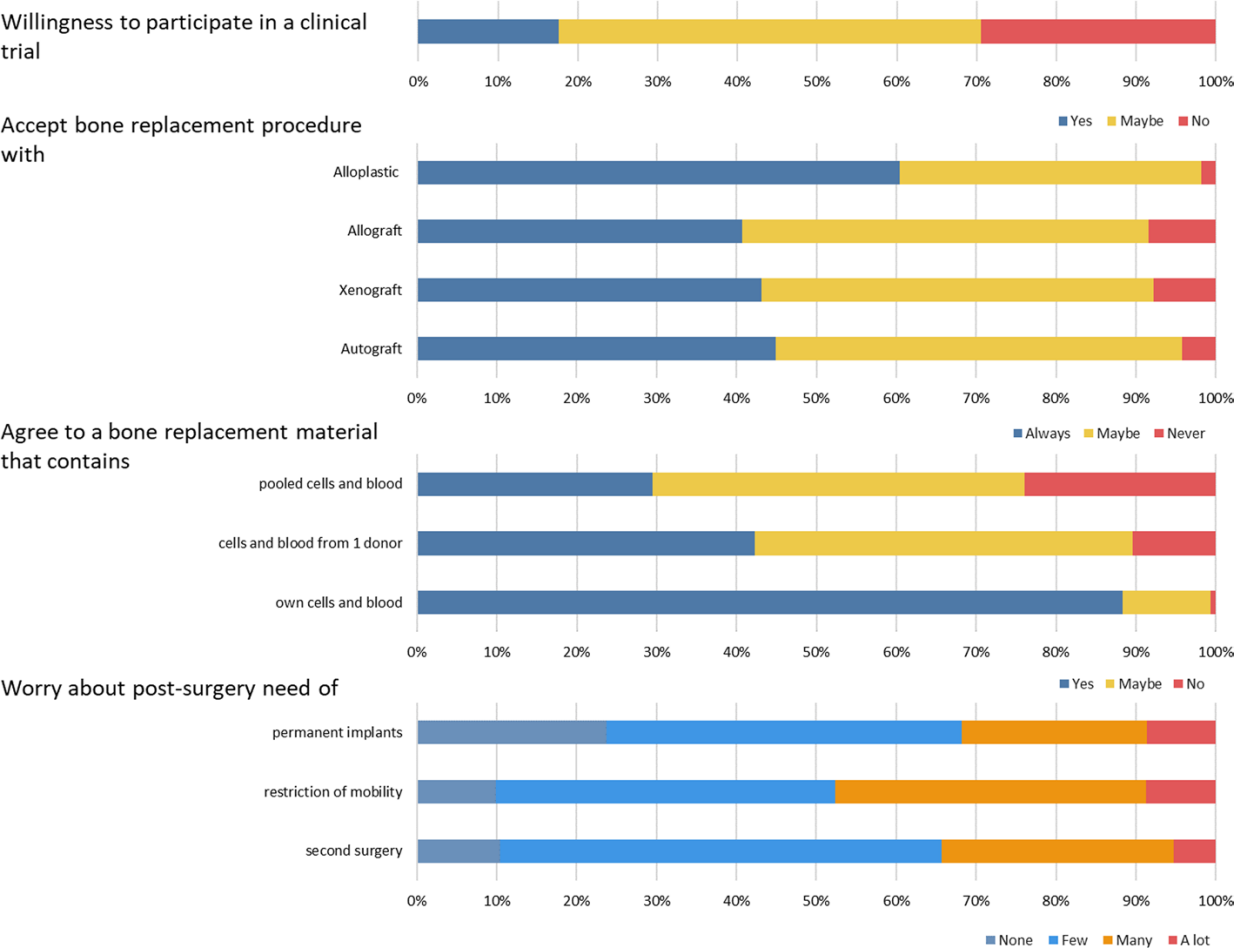
The rate of responses for the different questions from the survey

Presented with the option, more than half of the respondents (72%—yes and maybe responses) answered that they would take part in a clinical trial for new bone replacement materials solely previously tested in pre-clinical models (Fig. 2). When asked to imagine that surgery was needed to replace a large part of one of their bones, the respondents were more accepting of the use of alloplastic devices (61%—always responses) as bone replacements. The acceptance of allografts (bone from a human donor), xenografts (bone of animal origin), or autografts (bone taken from another body area) presented a similar frequency in the data (41–45%—always responses). Furthermore, most participants would agree to use an artificial material for a bone replacement that was mixed with their own blood and cells (87%—yes). However, there was less agreement with the use of a material mixed with pooled cells and blood from different individuals (29%—yes). When asked about post-surgical concerns in case of bone fractures, the respondents were more worried about mobility restriction and/or food intake restriction (48%—many or a lot) than the presence of permanent implants in their body (33%) or the need for a second surgery (35%).

**Fig. 3:**
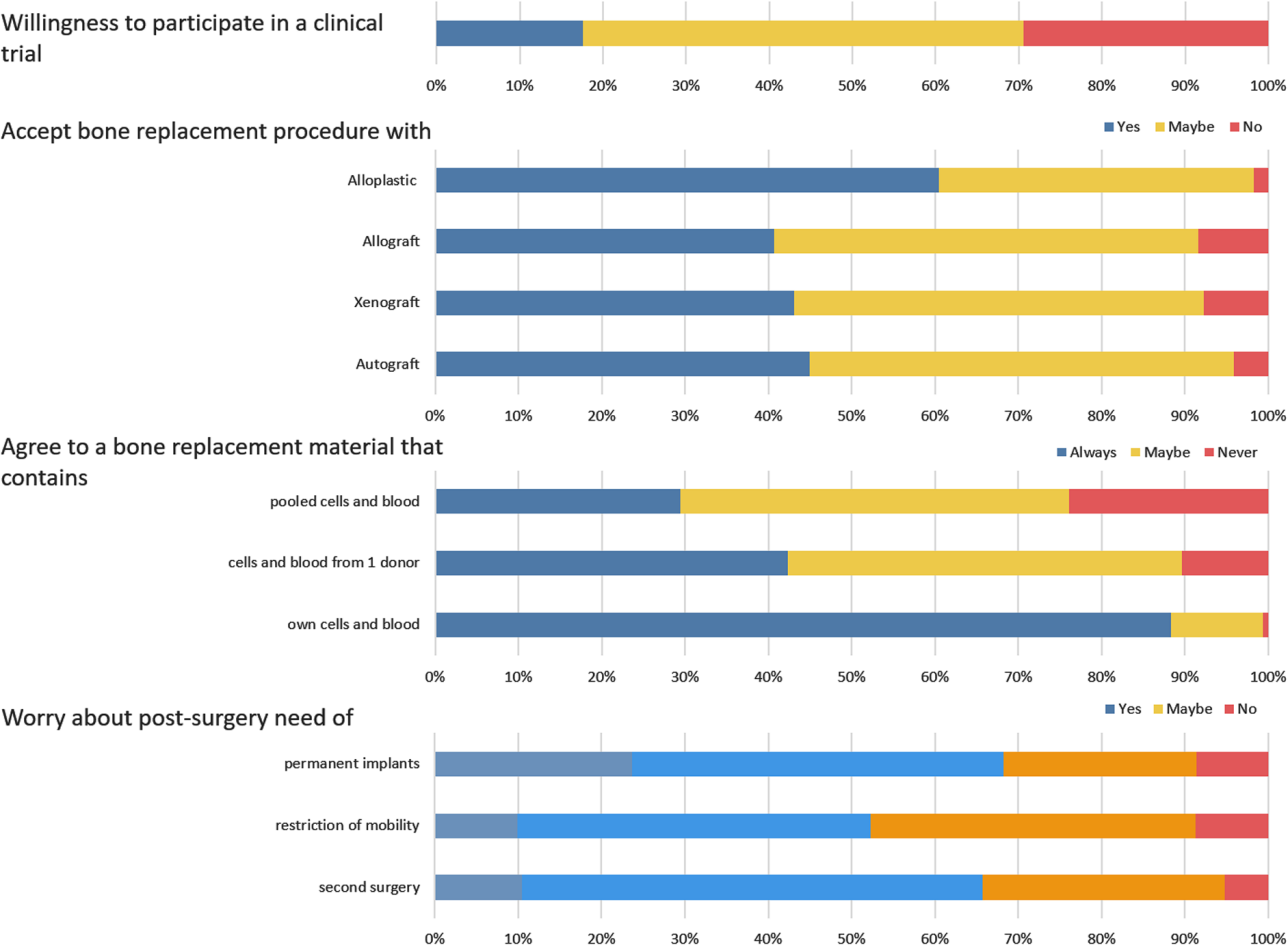
Representation of the responses influenced by generation and by education

The dichotomized responses to the queries about the bone replacement material and procedures were used to test for the influence of generation, gender, education, and dietary habits on the replies (Additional file 1: Table S2). The data showed that both generation and education influenced some of the responses (Fig. 3).

The data revealed that generation affected the participants accepting (yes/maybe responses) or rejecting (no responses) the participation in clinical trials (Chi-square, *p* = 0.03). People from older generations (Post-war/ Boomers/GenX) were more compliant with the participation in clinical trials. The generation also influenced the acceptance of alloplastic bone replacement materials (Fischer’s test, *p* = 0.02), showing that the younger generation (Millennials/GenZ) would be more accepting of alloplastic materials (Fig. 3).

The educational level also influenced individuals’ responses in two circumstances (*p* < 0.05). People with secondary education (no school certificate/elementary school/ middle school/high school) were less worried about the presence of permanent implants (none/few responses) post-surgery (*p* = 0.02). Furthermore, people who had post-secondary education (vocational training/technical school/university education/post-graduation) were less in agreement with receiving a bone replacement material (no responses) that contains pooled cells and blood (*p* = 0.03, Fig. 3).

## Discussion

The present work created a survey to help steer future developments in bone replacement materials and approaches. The questions were created in close collaboration between physicians and scientists aiming towards developing materials that would have better patient acceptance. The data have shown that overall patients trust scientifically developed biomaterials designed for bone replacement, as demonstrated by their willingness to participate in a clinical trial, their acceptance of alloplastic materials, and the none/few worries of the majority about the presence of permanent implants (67%). However, the data have also revealed the preferences of patients towards autologous sources of cells and blood to be used with a biomaterial, as well as how epidemiological differences, specifically generation and education influence people’s perspective on bone replacement materials and post-surgical worries.

The demographic analysis showed that there was a higher amount of younger generation respondents, which may be connected to the self-initiating method of the survey with the use of QR codes as an engagement tool, and the need for a smartphone or other digital device to scan it. QR codes were selected due to their ubiquity of use in Germany. Since the beginning of the COVID-19 crisis QR codes have been used as immunization certificates (European Union Digital COVID Certificate), for signing up for tests, as well as signing in when visiting shared spaces (e.g., museums, galleries, and restaurants). However, since Millennials and GenZ are more adept at technology [8], engaging with QR codes is more usual to them, which affected the demographics of the respondents. There were also more respondents with post-secondary education, which goes in line with the characteristics of the German population’s educational level [9]. Moreover, statistically, more females were flexitarian/vegetarian/vegans, which is a worldwide tendency [10–15] as women are more diet conscious than men [16–18]. The question about nutritional habits was implemented in the questionnaire to assess the correlation between peoples’ ideas about animal products' dietary consumption and the acceptance or rejection of xenografts. However, no correlation was found between the two in the present work.

Most of the respondents were willing to participate (yes and maybe responses) in a clinical trial with a newly developed material only tested in pre-clinical models, which is the opposite of what was found in a wide study of the Korean population [19]. Moreover, the older generation (GenX/Boomers/Post-war) of respondents were statistically more willing to participate in a clinical trial (*p* = 0.03), which is also contrary to what has been previously reported in a study profiling clinical trial participants [20]. Both differences in the present data to the literature might be an effect of the present study environment, a University hospital. Clinical research is one of the objectives of a University hospital, which is seen as positive by most potential patients [21]. Therefore, the mindset of the respondents might have been positively biased towards the possibility of receiving newly developed material in a clinical trial, as they were engaged in this survey inside a University hospital.

Furthermore, there was a higher acceptance of bone replacement procedures with alloplastic materials, which is in agreement with past findings in the literature [22, 23]. The acceptance was statistically higher in the younger generation of respondents. Biomaterials that contain cells or tissue (like blood) and can be applied in the body for therapeutic purposes are classified as tissue-engineered medical products. Most of the respondents were in agreement with the use of tissue-engineered medical products that contained their own cells or blood for bone replacement. This large amount of agreement might be due to the familiarity of patients with this kind of product. Germany is the European Union state with the highest number of clinical trials of tissue-engineered medical products [24]. Thus, the knowledge or even contact of German citizens with these kinds of biomaterials can facilitate their acceptance of tissue-engineered medical products. Moreover, most of the later-stage clinical trials being performed use autologous sources of cells [24]. This could also cause impact the opinions of respondents with higher educational levels, who were less in agreement with the use of pooled cells or blood (*p* = 0.03), as they might have more knowledge or contact with the autologous options and might fear the possible immune response to allogeneic cells.

When it came to the respondents’ post-surgery concerns, worries about the presence of permanent implants were influenced by educational level, and respondents with post-secondary education were statistically more worried about permanent implants (*p* = 0.02). That is in accordance with literature reports showing that the majority of patients and even the ones that suffer from complications caused by implant removal would choose to have implant removal surgery again [25]. Unfortunately, since the survey was composed of close-ended questions, it was not possible to evaluate the reasons for the respondents to worry about permanent implants, although the procedure entails a second surgery, which can impact a patient’s health. The lack of open-ended questions was the main limitation of the present study, as more information could have been gained from interviewing the participants. However, open-ended questions would have demanded more time for the participants for the questionnaire, which might have impacted the total number of participants.

The present study has revealed the perspective of the participants about materials that could be potentially used for their bone regeneration. The trend in translational research is to involve patients as stakeholders to support the development of new therapies [26]. This also includes families and caregivers, whose experiences can enrich the project from the inception phase up until the publication of findings [26].

## Conclusions

In summary, patients appear to be open to surgical approaches that involve the implantation of biomaterials for the regeneration of bone. A preference exists for strategies that use the patient’s own blood and/or tissue in comparison with strategies that use blood pools from different donors. Moreover, patients are concerned about the strategies based on permanent implants which stay in the body which indicates a need to develop therapeutic strategies based on resorbable materials. Furthermore, patients’ views on materials and surgical techniques are influenced by both educational level and generation. Finally, the knowledge gained in this study could guide the development of bone biomaterials taking into account patients’ perspectives.

### Methodology

The survey was planned as a self-initiating cross-sectional query aimed at people seeking treatment at the university hospital environment of the Charité Berlin. The survey was designed with the input of different stakeholders in the field of tissue engineering; namely, physicians in the fields of trauma, orthopedics, and maxillofacial surgery, senior and junior researchers in the fields of biology, molecular biology, veterinary medicine, bioengineering, and material sciences, as well as doctoral students in the field regenerative therapies. Their experience with the technology as well as their concerns about the patient’s general view of the materials was what ignited the design of the questionnaire. Short and direct questions were formulated to improve patient understanding and easiness of reply. Special attention was paid to keeping the language understandable for people without a medical education. A testing round with 5 people from different age groups and educational backgrounds was undertaken to ensure that the language was inclusive and non-offensive. Another test using different Smartphone devices was also implemented, which was used as an assurance that the survey was adaptable to different screens.

The survey was collected and managed using Research Electronic Data Capture (REDCap) tools, which was hosted at Charité—Universitätsmedizin Berlin [27]. REDCAp is a web-based software platform that supports anonymized secure data collection for research purposes. No identifying data were asked from the participants to protect their privacy rights. A timestamp of the moment when the participant started replying to the survey was used to organize the samples in numerical order.

The questionnaire was designed to take about 4 min of the participants’ time. To do so, a total of 15 close-ended questions in German, their native language, were used (original survey in Additional file 1: Table S1). Four questions were directed toward the participant’s profile, explicitly about their generation, gender, education, and nutritional habits. For the other eleven questions, rate scale answers with either 3 or 4 different possible choices were developed. From those, eight questions were focused on the participants’ willingness, acceptance, and agreement to receive different and new bone replacement materials (Table 1). The other three questions were aimed at their worries about the post-surgical consequences that can arise due to bone replacement surgery (Table 1).

**Table 1:** Rate scale questions and answers

Questions	Levels
Willingness to participate in a clinical trial	Yes
	Maybe
	No
Accept a bone replacement procedure with	
Autograft	Always
	Maybe
	Never
Xenograft	Always
	Maybe
	Never
Allograft	Always
	Maybe
	Never
Alloplastic	Always
	Maybe
	Never
Agree to a bone replacement material that contains	
Own cells and blood	Yes
	Maybe
	No
Cells and blood from 1 donor	Yes
	Maybe
	No
Pooled cells and blood	Yes
	Maybe
	No
Worry about the post-surgery need of	
Second surgery	None
	Few
	Many
	A lot
Restriction of mobility	None
	Few
	Many
	A lot
Permanent implants	None
	Few
	Many
	A lot

The survey was distributed as a quick response (QR) code advertised in posters which contained a provocative question (“How should we heal your bones?”) with a call for action (“Scan me”) throughout the waiting areas of the Campi of the Charité University Hospital Berlin, at both emergency rooms and outpatient clinics. Therefore, any literate person who spoke German and had a Smartphone with a camera could participate.

Survey data were collected from May until December 2022. A total of 198 people engaged with the QR code, and from those 187 responded to the questions either partially or completely. A descriptive analysis of the respondents was performed using pivot tables. Further contingency tables were created with categorical dichotomous variables to group the population and responses for statistical analysis. To compare the observed number of subjects in each related category either Pearson’s chi-square test or Fischer’s test was used (GraphPad Prism 9.5.0) with a 95% confidence level (*p* < 0.05).

### Supplementary Information

**Additional file 1: Table S1.** The original questionnaire was applied in plain German language so that it could be understood and answered by a diverse population of survey participants. **Table S2.** Dichotomized data arranged in contingency tables to test the influence of different respondents characteristics, like generation (Post-war/Boomers/GenX or Milenials /GenZ), gender (female/diverse or male), education level (post-secondary education (Vocational training/Technical school/University education/Post-graduation) or until secondary education (No school certificate/Elementary school/Middle school/High school)), and nutrition (Flexitarian/Vegetarian /Vegan or Omnivore) on the responses to the queries about bone replacement material and procedures. Statistical significance tested using either Pearson’s chi-square test or Fischer’s test (p<0.05 represented in green).

## Data Availability

All data generated or analyzed during this study are included in this published article [and its Additional file 1].
