# Supplementary material for: Patients ‘ perspectives on bone replacement materials in a German university hospital setting
Source: Biomed Eng Online. 2023 Aug 28;22:84. doi: 10.1186/s12938-023-01147-2 (PMC10464219; doi:10.1186/s12938-023-01147-2)
Supplement: Supplementary file 1 — Additional file 1: Table S1. The original questionnaire was applied in plain German language so that it could be understood and answered by a diverse population of survey participants. Table S2. Dichotomized data arranged in contingency tables to test the influence of different respondents characteristics, like generation (Post-war/Boomers/GenX or Milenials /GenZ), gender (female/diverse or male), education level (post-secondary education (Vocational training/Technical school/University education/Post-graduation) or until secondary education (No school certificate/Elementary school/Middle school/High school)), and nutrition (Flexitarian/Vegetarian /Vegan or Omnivore) on the responses to the queries about bone replacement material and procedures. Statistical significance tested using either Pearson’s chi-square test or Fischer’s test (p<0.05 represented in green). [file 12938_2023_1147_MOESM1_ESM.pdf]

Additional information:

Patients' perspective on bone replacement materials in a German university hospital setting

Ana Prates Soares, Heilwig Fischer, Vincenzo Orassi, Max Heiland, Sara Checa, Katharina Schmidt-Bleek, Carsten Rendenbach

**1.**	**In welchem Zeitraum sind Sie geboren?**<br>a. 1997-2004 b. 1981-1996 c. 1965-1980 d. 1946-1964 e. 1928-1945
**2.**	**Welches Geschlecht haben Sie?**<br>a. Weiblich b. Männlich c. Divers
**3.**	**Was ist ihr höchster Bildungsabschluss?**<br>a. Kein Schulabschluss b. Grund-, Hauptschulabschluss c. Realschule – mittlere Reife d. Gymnasium – Abitur e. Abgeschlossene Ausbildung f. Fachhochschulreife g. Hochschulabschluss h. Promotion
**4.**	**Wie ernähren Sie sich?**<br>a. Omnivore Ernährung (keine Einschränkungen) b. Bewusste Ernährung (ich esse selten Fleisch) c. Vegetarier d. Vegan
**5.**	**Ein neues Knochenersatzmaterial hat vielversprechende Ergebnisse im Tierversuch gezeigt. Wären Sie bereit, sich dieses Knochenersatzmaterial implantieren zu lassen, auch, wenn noch keine Ergebnisse aus Studien am Menschen vorliegen?**<br>a. Ja b. Nein c. Vielleicht
**6.**	**Stellen Sie sich vor, Ihr/e Arzt/Ärztin teilt Ihnen mit, dass eine Operation mit Einbringung einer Platte notwendig ist, um einen Knochenbruch zu versorgen. Wie viele Sorgen machen Sie sich um die folgenden Aspekte?**<br>**a. Notwendigkeit einer zweiten Operation**<br>i. Keine ii. Ein wenig iii. Viele iv. Sehr<br>**b. Zeitlich begrenzte Einschränkung der Beweglichkeit oder Nahrungsaufnahme**<br>i. Keine ii. Ein wenig iii. Viele iv. Sehr<br>**c. Permanente Implantate (Metall oder Keramik) in meinem Körper**<br>i. Keine ii. Ein wenig iii. Viele iv. Sehr
**7.**	**Stellen Sie sich vor, Ihr/e Arzt/Ärztin teilt Ihnen mit, dass eine Operation mit Einbringung einer Platte notwendig ist, um einen Knochenbruch zu versorgen. Wie viele Sorgen machen Sie sich um die folgenden Aspekte?**<br>**a. Es wird Ihnen selbst Knochen von einer anderen Körperregion entnommen und verwendet:**<br>i. Immer ii. Niemals iii. Vielleicht<br>**b. Knochen tierischen Ursprungs (verarbeitet und ohne Risiken für Leib und Leben) wird verwendet:**<br>i. Immer ii. Niemals iii. Vielleicht<br>**c. Knochen eines menschlichen Spenders (verarbeitet und ohne Risiken für Leib und Leben) wird verwendet:**<br>i. Immer ii. Niemals iii. Vielleicht<br>**d. Künstliches Knochenmaterial (verarbeitet und ohne Risiken für Leib und Leben) wird verwendet:**<br>i. Immer ii. Niemals iii. Vielleicht

8. Wenn das künstliche Knochenmaterial Zellen und/oder Blut enthalten würde, welcher der folgenden Optionen würden Sie zustimmen?

a. **Zellen und Blut stammen von Ihnen selbst und werden mit dem Knochenmaterial vermischt**

i. Ja ii. Nein iii. Vielleicht

b. **Zellen und Blut stammen von einem menschlichen Spender und werden mit dem Knochenmaterial vermischt**

i. Ja ii. Nein iii. Vielleicht

c. **Zellen und Blut stammen von verschiedenen menschlichen Spendern und werden mit dem Knochenmaterial vermischt**

i. Ja ii. Nein iii. Vielleicht

Supplementary table 1: The original questionnaire was applied in plain German language so that it could be understood and answered by a diverse population of survey participants.

		GenX / Boomers / Post-war	Milenials / GenZ	Female/ Diverse	Male	Post-secondary education	Until secondary education	Flexitarian/ Vegetarian /Vegan	Omnivore
Clinical trial participation (n=187)	**No**	12	43	31	24	38	17	29	26
	**Yes/maybe**	51	81	65	67	84	48	71	61
	**p=**	0.03		0.37		0.48		0.89	
Worry about 2nd operation (n=172)	**Few / None**	35	77	61	51	75	37	65	47
	**Many / A lot**	24	36	30	30	41	19	29	31
	**p=**	0.25		0.58		0.86		0.22	
Worry about functional limitations (n=171)	**Few / None**	32	57	44	45	65	24	49	40
	**Many / A lot**	27	55	47	35	51	31	45	37
	**p=**	0.68		0.30		0.13		0.98	
Worry about permanent implants (n=173)	**Few / None**	43	75	59	59	73	45	65	53
	**Many / A lot**	17	38	32	23	44	11	29	26
	**p=**	0.48		0.32		0.02		0.77	
Acceptance of Autograft (n=167)	**Never**	3	4	3	4	5	2	3	4
	**Always / Maybe**	56	104	86	74	108	52	91	69
	**p=**	0.67		0.57		0.83		0.46	
Acceptance of Xenograft (n=167)	**Never**	7	6	10	3	11	2	7	6
	**Always / Maybe**	52	102	79	75	102	52	87	67
	**p=**	0.15		0.08		0.17		0.85	
Acceptance of Allograft (n=167)	**Never**	7	7	9	5	10	4	8	6
	**Always / Maybe**	52	101	80	73	103	50	86	67
	**p=**	0.23		0.39		0.75		0.95	
Acceptance of Alloplastic (n=167)	**Never**	3	0	1	2	2	1	2	1
	**Always / Maybe**	56	108	88	76	111	53	92	72
	**p=**	0.02		0.48		0.97		0.71	
Agree to material containing own cells (n=163)	**No**	1	0	0	1	0	1	1	0
	**Yes/Maybe**	56	106	88	74	111	51	91	71
	**p=**	0.17		0.28		0.14		0.38	
	**No**	7	10	11	6	14	3	13	4

Agree to material containing cells/blood from a single donor (n=163)	**Yes/Maybe**	50	96	77	69	97	49	79	67
	**p=**	0.57		0.35		0.18		0.08	
Agree to material containing cells/blood from pooled donors (n=163)	**No**	17	22	25	14	32	7	26	13
	**Yes/Maybe**	40	84	63	61	79	45	66	58
	**p=**	0.20		0.15		**0.03**		0.14	

Supplementary Table 2: Dichotomized data arranged in contingency tables to test the influence of different respondents characteristics, like generation (Post-war/Boomers /GenX or Milenials /GenZ), gender (female/diverse or male), education level (post-secondary education (Vocational training/Technical school/University education/Post-graduation) or until secondary education (No school certificate/Elementary school/Middle school/ High school)), and nutrition (Flexitarian/Vegetarian /Vegan or Omnivore) on the responses to the queries about bone replacement material and procedures. Statistical significance tested using either Pearson's chi-square test or Fischer's test ($p<0.05$ represented in green).
